# Comment on: The temporal relationship between cancer and adult onset anti-transcriptional intermediary factor 1 antibody–positive dermatomyositis: Reply

**DOI:** 10.1093/rheumatology/kez329

**Published:** 2019-08-03

**Authors:** Alexander Oldroyd, Jamie C Sergeant, R Paul New, Neil J McHugh, Zoe Betteridge, Janine A Lamb, William E Ollier, Robert G Cooper, Hector Chinoy

**Affiliations:** 1 Centre for Musculoskeletal Research, University of Manchester, Manchester, UK; 2 NIHR Manchester Biomedical Research Centre, Central Manchester NHS Foundation Trust, Manchester Academic Health Science Centre, Manchester, UK; 3 Centre for Biostatistics, Manchester, UK; 4 Centre for Epidemiology Versus Arthritis, University of Manchester, Manchester, UK; 5 MRC/ARUK Centre for Integrated Research into Musculoskeletal Ageing, University of Liverpool, Liverpool, UK; 6 Department of Pharmacy and Pharmacology, University of Bath, Bath, UK; 7 Royal National Hospital for Rheumatic Diseases, Royal United Hospitals Foundation Trust, Bath, UK; 8 Division of Population Health, Health Services Research and Primary Care, Manchester, UK; 9 Centre for Integrated Genomic Medical Research, University of Manchester, Manchester, UK; 10 Department of Rheumatology, Aintree University Hospital, Liverpool, UK; 11 Department of Rheumatology, Salford Royal NHS Foundation Trust, Salford, UK

Sir, We read with great interest the letter by Korsten *et al.* [1] in response to our article on cancer risk in anti-transcriptional intermediary factor 1 antibody (anti-TIF1-Ab)-positive DM [2].

Korsten *et al.* [1] highlighted the clinical relevance of our findings, in particular the need for focussed cancer screening in anti-TIF1-Ab-positive patients, tailored to a patient’s disease duration (highest incidence within 2.5 years after DM onset), age (no cancers observed in those <39 years of age) and gender (high incidence of ovarian and breast cancer). Korsten *et al.* also quite rightly pinpoint the important question of how cancer screening should be carried out; in particular, what modalities should be employed and how frequently this should occur. Further, they raise the important question of whether such screening may even impact upon overall prognosis at all.

The case that Korsten *et al.* describe clearly illustrates the importance of vigilance of cancer-related symptoms and repeated cancer screening within the 3 years after DM onset. Unfortunately, the body of empirical evidence on the utility of screening is limited, with results of relatively small observational studies forming the basis for recommendations.

Selva-O’Callaghan *et al.* [3] compiled the relevant evidence in a 2018 review. A clinically useful flowchart with recommendations for the frequency of cancer screening was developed and reported, guided primarily by a patient’s autoantibody status; of note, annual screening up to 5 years after DM onset was advised for anti-TIF1-Ab-positive patients. The Epidemiological Useful Clinical–Laboratory–Imaging Development Screening (EUCLIDES) approach was also described, providing overall guidance on focussed cancer screening in all forms of idiopathic inflammatory myopathy (IIM). The use of PET/CT was advocated, along with whole-body MRI, as a means to identify occult malignancy and delineate the entire burden of myositis.

Trials of cancer screening approaches in large IIM cohorts may not be feasible, due in part to the low incidence of the disease. However, data from large international IIM registries, along with careful statistical analysis, could potentially provide answers to specific questions, such as the optimum time of cancer screening, impact upon overall survival and utility of specific investigation modalities. Further, the utility of novel technologies in IIM-related cancer should be investigated, such as ‘liquid biopsy’, which allows the detection of circulating tumour cells [4]. This may provide a means to detect subclinical cancer at a stage early enough to confer improved survival. Evidence-based guidelines with expert recommendations are certainly required. Such guidance is currently in development via the International Myositis Assessment and Clinical Studies Group (IMACS).
